# Presence of intestinal *Mycobacterium avium *subspecies *paratuberculosis *(MAP) DNA is not associated with altered MMP expression in ulcerative colitis

**DOI:** 10.1186/1471-230X-11-34

**Published:** 2011-04-08

**Authors:** Timo Rath, Martin Roderfeld, Sonja Blöcher, Annika Rhode, Tina Basler, Ömer Akineden, Amir Abdulmawjood, Jörg M Halwe, Ralph Goethe, Michael Bülte, Elke Roeb

**Affiliations:** 1 Department of Gastroenterology, Medical Clinic II, Justus Liebig University, Paul-Meimberg-Strasse 5, 35392 Giessen, Germany; 2 Institute for Microbiology, Centre for Infectious Medicine, Veterinarian Medical Academy Hannover, Bischofsholer Damm 15, 30173 Hannover, Germany; 3 Institute of Veterinary Food Science, Faculty of Veterinary Medicine, Justus Liebig University, Frankfurter Strasse 92, 35392 Giessen, Germany; 4 Department of Internal Medicine, University Hospital Stavanger, 4011 Stavanger, Norway; 5 Department of Internal Medicine, Stord Hospital, 5416 Stord, Norway

## Abstract

**Background:**

*Mycobacterium avium *subspecies *paratuberculosis *(MAP) is suspected to be a causative agent in human Crohn's disease (CD). Recent evidence suggests that pathogenic mycobacteria and MAP can induce the expression of Matrix Metalloproteinases (MMP), which are the main proteases in the pathogenesis of mucosal ulcerations in inflammatory bowel disease (IBD). Within this study we assessed the prevalence of intestinal MAP specific DNA in patients with Crohn's disease, ulcerative colitis (UC), and healthy controls. We further analysed regulation patterns of MMPs in mucosal tissues of UC patients with and without intestinal MAP DNA detection.

**Methods:**

Colonic biopsy samples were obtained from 63 Norwegian and German IBD patients and 21 healthy controls. RNA was quantified by quantitative real-time polymerase chain reaction (PCR) to study MMP gene expression in both pathological and healthy mucosal specimens. The presence of MAP DNA in colonic mucosa was examined using MAP specific PCR.

**Results:**

MAP DNA was detected in 20% of UC patients and 33% of healthy controls but only in 7% of patients with CD. UC patients treated with corticosteroids exhibited a significantly increased frequency of intestinal MAP DNA compared to those not receiving corticosteroids. Expression of MMP-1, -2, -7, -9, -13, -19, -28 and TNF-α did not differ between UC patients with presence of intestinal MAP DNA compared to those without. MMP-2, MMP-9 and MMP-13 were significantly decreased in UC patients receiving corticosteroids.

**Conclusions:**

The presence of intestinal MAP specific DNA is not associated with altered MMP expression in UC *in vivo*. Corticosteroids are associated with increased detection of intestinal MAP DNA and decreased expression of certain MMPs. Frequent detection of MAP DNA in healthy controls might be attributable to the wide environmental distribution of MAP and its presence in the food-chain.

## Background

*Mycobacterium avium *strains are widely distributed in the environment and inhabit animal and human intestines. *Mycobacterium avium *subsp. *paratuberculosis *(MAP) is the causative agent of Johne's disease, a chronic granulomatous inflammation of the intestines in ruminants like dairy cows and many other species, including primates [1].

Crohn's disease (CD) and ulcerative colitis (UC) represent the major forms of human idiopathic inflammatory bowel disease (IBD). Accumulating evidence suggests that CD results from an excessive mucosal immune response towards intestinal microbes in a genetically susceptible host [2]. Since the initial description of clinical similarities between Crohn's disease and Johne's disease in cattle in 1913 [3], it has been hypothesized that MAP might represent a causative agent in CD. Since then, a number of studies and meta-analyses have reported about a more frequent detection of MAP in patients with CD than in controls [4-7]. Nevertheless, it is still a matter of controversy whether MAP represents an etiologic factor for CD or rather a secondary invader of inflamed intestinal mucosa [6,8,9]. So far, *in vitro *studies have identified increased TNF-α levels in mucosal organ culture supernatants from MAP positive CD patients as well as an increased T-cell proliferation upon incubation of peripheral blood mononuclear cells from CD patients with MAP as pathogenic mechanisms and cellular responses [10,11].

Matrix Metalloproteinases (MMPs) are a family of Zn^2+^-dependent endopeptidases that are considered to be the most potent proteases in the turnover of the extracellular matrix (ECM) [12]. In addition to their capability of degrading virtually all protein components of the ECM, MMPs regulate a variety of non-matrix substrates such as chemokines, cytokines and growth factors and influence the function and migration of inflammatory cells [12]. They are considered to be the predominant proteases in the pathogenesis of mucosal ulcerations associated with IBD [13-15]. Furthermore, evidence suggests that MMPs are upregulated upon infection with pathogenic mycobacteria and MAP thereby leading to the inflammatory tissue changes associated with mycobacterial infections [16-18].

Within the present study we determined the prevalence of MAP DNA in biopsy samples of patients with UC and CD as well as in patients without IBD using highly sensitive and MAP specific PCRs. To analyse a potential regulation of MMP expression by MAP *in vivo*, we further assessed the colonic expression of a broad MMP spectrum in UC patients with and without intestinal MAP DNA detection.

## Methods

### Patients and biopsy samples

From June 2007 to July 2008, biopsy samples were obtained from 63 German (n = 21) and Norwegian (n = 42) patients with IBD and from 21 German non-IBD patients during diagnostic colonoscopies. Diagnosis of IBD was confirmed clinically and histologically. Biopsies were taken from areas that macroscopically showed the highest degree of inflammation and were in closest proximity to those biopsies taken for histopathological examinations. Additionally samples were taken in areas with milder signs of inflammation or normally (i.e. unaffected) appearing mucosa. The Mayo endoscopic subscore was utilized for the macroscopic classification of the degree of inflammation [19].

Biopsy samples from the German IBD patients (n = 21) and the German non-IBD controls (n = 21) for MAP culture were placed in a 1.5-ml sterile screw-cap reaction tube containing 0.5 ml of sterile 0.85% saline and immediately transferred to the laboratory for further investigations. Samples for cultural investigation were not frozen at any time. All biopsies scheduled for MMP analysis were immediately cryopreserved in liquid nitrogen and stored at -80°C until examination. Biopsies underwent a thorough histopathological examination and were classified as "no", "low-grade", or "high-grade" intestinal neoplasia. These examinations were carried out by an experienced pathologist who was neither involved in the study protocol nor aware of the particular MMP measurements. All patients were informed about the study and gave their written consent. In Norway the study was approved by the ethics committee, the data protection commissioner, and the representative for patients' interests and safety (*Pasientvernombudet*) of the University Bergen. The Norwegian health authority (*Helsedirektorat*) authorised both, the study and the shipping of the tissue samples from Stavanger, Norway, to Giessen, Germany. In Giessen the study was approved by the local ethic committee (No 75/2009).

### Culture of biopsy specimens for MAP detection

The biopsy specimens from 21 German patients with IBD and 21 non-IBD patients was decontaminated when fresh and subsequently cultured in separate MGIT culture tubes at 37°C for up to 18 months. Briefly, 0.5 ml of NaOH-N-acetyl-L-cysteine (Merck, Darmstadt, Germany) was added to each sample in 0.5 ml of saline, and the mixture was incubated at room temperature for 20 min with occasional mixing by inversion. The samples were then centrifuged (10,000 × *g *for 10 min), and the supernatant was removed. The pellet was resuspended in 0.5 ml of PBS (14.6 mM KH_2_PO_4_, 2 mM Na_2_HPO_4 _[pH 6.8]) and transferred to the tube containing 4.5 ml of MGIT medium (Becton Dickinson, Heidelberg, Germany) supplemented with 10% oleic acid-albumin-dextrose-catalase (OADC), PANTA (40 U of polymyxin B per ml, 4 μg of amphotericin B per ml, 16 μg of nalidixic acid per ml, 4 μg of trimethoprim per ml, 4 μg of azlocillin per ml [final concentrations]), and 2 μg of mycobactin J (Allied Monitor, Fayette, MO, USA) per ml. After incubation at 37°C for 12 and 52 weeks, the cultures were mixed and 0.5 ml was taken aseptically for testing by the MAP specific PCR as described below. MGIT cultures with visible growth or with a positive PCR result were subcultured onto Herrold's Egg Yolk Medium with ANV (Ampothericin B, Nalidixic acid, Vancomycin) and Mycobactin J and incubated at 37°C for up to 12 months. Biopsy samples from Norwegian patients (n = 42) were processed for direct specific PCR testing.

### DNA extraction and PCR analysis for MAP detection

The DNA of the biopsy samples and MGIT cultures after 12 and 52 weeks was extracted using a modified protocol of the DNeasy Blood and Tissue Kit (Qiagen, Hilden, Germany) for the extraction of DNA from Gram-positive bacteria. As a modification to the protocol of the DNeasy Blood and Tissue kit, samples were processed in a spin/rotation instrument for cell lysis (FastPrep-120), with a speed and time setting of 6 and 45 seconds, respectively.

For the molecular detection of MAP, two PCR systems were set up in parallel, a triplex real time-PCR targeting MAP specific genes F57 and IS*Mav2 *and an internal amplification control (IAC) [20], and a nested PCR [21] with IS*900 *oligonucleotide primers. The PCR reaction mixture for the nested PCR (50 μl) contained 1 μl primer 1 (10 pmol/μl), 1 μl primer 2 (10 pmol/μl), 1 μl dNTP (10 mmol, Roche), 5 μl 10 × thermophilic-buffer (Applied Biosystems, Darmstadt, Germany), 0.5 μl MgCl_2 _(25 mM, Applied Biosystems), 0.5 μl Ampli *Taq *Gold DNA polymerase (5 U/μl, Applied Biosystems) and 36 μl double-distilled water. Finally, 5 μl of the DNA preparation was added to each reaction tube. The triplex real time-PCR assay was performed as previously described [20]. The fluorescent data were generated by TaqMan^®^_mgb _probes applied on the ABI Prism^® ^7000 Sequence Detection System according to the instructions of the supplier. Briefly, the 50 μl PCR mixture for the triplex real time-PCR assay consisted of 25 μl of the 2xqPCR MasterMix Plus w/o uracil N-glycosylase (UNG, Eurogentec, Seraing, Belgium), 1 μl of each primer (F57, IS*Mav2*), 2 μl of each of the fluorogenic probes (F57, IS*Mav2*, IAC), 1.5 μl of the IAC, 8.5 μl of double-distilled water and a 5-μl aliquot of the DNA sample. The PCRs were performed in a 96well plate format on the ABI Prism^® ^7000 Sequence Detection System (Applied Biosystems). Thermal cycling conditions comprised a Hot Start DNA Polymerase activation at 95°C for 10 minutes, 50 cycles of denaturation at 95°C for 15 seconds, and an annealing and extension at 60°C for 1 minute. Each measurement was performed in duplicate, and the threshold cycle (Ct) was determined. Primer sequences and fluorogenic probes for MAP detection are presented in Table 1.

**Table 1:** Oligonucleotide primers and fluorogenic probes for MAP detection

Designation	Sequence	Reference
**F57 forward**	5'-FTA CGA GCA CGC AGG CAT TC-3'	Schönbrücher et al. [20]
**F57 reverse**	5'-CGG TCC AGT TCG CTG TCA T-3'	
**F57 TaqMan^®^_mgb_-probe**	VIC -CCTGACCACCCTTC-MGB	
**F57 TaqMan^®^_mgb_-IAC**	NED-CGAGTTACATGATCCC-MGB	
		
**IS*Mav2 *forward**	5'-CGG CAA AAT CGA GCA GTT TC-3'	
**IS*Mav2-*R reverse**	5'-TGA GCC GGT GTG ATC ATC TTT-3'	
**IS*Mav2 *TaqMan^®^_mgb_-probe**	FAM-CGC TGA GTT CCT TAG-MGB	
		
**TJ1 forward**	5'-GCT GAT CGC CTT GCT CAT-3'	Bull et al. [21]
**TJ2 reverse**	5'-CGG GAG TTT GGT AGC CAG TA-3'	
**TJ3 forward**	5'-CAG CGG CTG CTT TAT ATT CC-3'	
**TJ4 reverse**	5'-GGC ACG GCT CTT GTT GTA GT-3'	

### RNA Purification, cDNA Synthesis and RT-PCR for MMP analyses

Biopsy samples were homogenized with a polytron homogenizer (Kinematica, Luzern, Switzerland) and total cellular RNA was extracted from shock-frozen single biopsies with RNeasy Kit (Qiagen, Hilden, Germany) according to the manufacturer's instructions. First-strand cDNA was synthesized from 1 μg DNA-free total RNA using the oligo-dT-primers and the first-strand cDNA synthesis kit for RT-PCR (Roche Diagnostics, Mannheim, Germany). Real time-PCR was performed using Platinum SYBR Green qPCR Kit (Invitrogen, Karlsruhe, Germany) according to the manufacturer's protocol. Real time-PCR of each gene-specific primer pair was optimized prior to the experiment to confirm the absence of any non-specific amplification product. Primers were purchased from Eurofins (Ebersberg, Germany) and primer sequences are presented in Table 2.

**Table 2:** SYBR Green Real-time qPCR Primer sequences.

Gene	Primer Sequence	GenBank **AccessionNo**.
**human MMP-1**	Fw: 5'-AAG TTG AAA AGC GGA GAA ATA G-3' Rev: 5'-TTT CAA TCC TGT AGG TCA GAT G-3'	NM 002421
**human MMP-2**	Fw: 5'-GGC AGA CAT CAT GAT CAA CT-3' Rev: 5' TGC TGT CAT AGG ATG TG-3'	NG 008989
**human MMP-7**	Fw: 5'-AGT TTA GAA GCC AAA CTC AAG G-3' Rev: 5'-GCG GTA AGT CTC GAG TAT ATG-3'	NM 002423
**human MMP-9**	Fw: 5'-TTG ACA GCG ACA AGA AGT GG-3' Rev: 5'-GTA CAT AGG GTA CAT GAG CG-3'	NG 011468
**human MMP-13**	Fw: 5'-GCA GTC TTT CTT CGG CTT AG-3' Rev: 5'-GGA GTT ACA TCG GAC CAA AC-3'	NM 002427
**human MMP-19**	Fw: 5'-CTT TCA AGG GGG ACT ATG TG-3' Rev: 5'-TAT TCA GCT TCT TGG GGA AG-3'	NM 002429
**human MMP-28**	Fw: 5'-GAG GCA TTC CTA GAG AAG TAC G-3' Rev: 5'-CTA GCA AAC AAG TCA CTG ATC C-3'	NM 024302
**human TNF-α**	Fw: 5'-CAT GTT GTA GCA AAC CCT CA-3' Rev: 5'-CTT GGT CTG GTA GGA GAC G-3'	NM 000594
**human 18sRNA**	Fw: 5'-GAT CAG ATA CCG TCG TAG TTC C-3' Rev: 5'-TAT CAA TCT GTC AAT CCT GTC C-3'	NR 003286

qRT-PCR was performed on the Mx3000P (Stratagene, La Jolla, USA) by using 3-stage program parameters as follows: 1) 10 minutes at 96°C, 2) 40 cycles of 10 seconds at 95°C, 30 seconds at 57°C, and 30 seconds at 73°C, 3) 10 minutes at 73°C. The specificity of the PCR was confirmed by examination of the dissociation reaction plot subsequent to qRT-PCR. PCR products were separated on a 1.5% TAE agarose gel and visualized by staining with ethidium bromide to confirm the appearance of a single band of the correct molecular size. Furthermore, specificity of the PCR reactions and the utilized primers for MMP analyses was verified in sequencing analyses. qRT-PCR data were analysed using the ΔΔCt model [22].

### Statistical analysis

Prior to more sophisticated statistical calculations, normal distribution of the data was tested using the Kolmogorov-Smirnov test and visualization of histograms. Failing to meet criteria for normal distribution all data were analysed using non-parametric tests. To compare MAP DNA positive detection rates in the three groups CD, UC, and control patients, Fisher's exact test was applied. Individual MMP expression in UC affected colonic mucosa was referred to the respective expression in corresponding healthy mucosa of the same individual in order to account for interindividual differences in colonic MMP expression. Results of MMP expression in relation to the MAP infection status are presented in box-and-whisker-plots. The upper hinge of the box represents the 75th percentile; the lower hinge represents the 25th percentile. The line in the box indicates the median value of the data. The ends of the vertical lines represent the minimum and maximum. Outliers (□) have a distance from 1.5 to 3 box lengths from the upper or lower hinge. Extreme values (Δ) have a distance of >3 box lengths from the upper or lower hinge. Interindividual comparisons of MMP expression were made using Mann-Whitney-U test. A two-sided *P *< 0.05 was considered significant. Statistical analysis was performed with SPSS 17.0 (SPSS Inc, Chicago, Ill).

## Results

### Demographic and clinical data of the patient cohort

Intestinal biopsies were obtained during routine colonoscopies from adult patients with CD (n = 14), UC (n = 49) and controls without IBD or other inflammatory disorders within the intestines (n = 21). Diagnosis of CD and UC was confirmed in accordance with the current guidelines based on histopathological examination and clinical findings. Table 3 summarizes the clinical data of these patients.

**Table 3:** Demographical and clinical data of the IBD patient cohort

	UC (n = 49)	CD (n = 14)
**Age (years)**		
- mean +/-SD	39.6 +/- 13.5	32.4 +/- 13.1
- range	19-74	20-68
**Sex [male/female (n)]**	26/23	7/7
**Disease manifestation UC (n)**		
- rectum	4	
- rectosigmoid	19	
- including C. descendens (leftsided colitis)	9	
- pancolitis	17	
**Disease manifestation CD (n)**		
- terminal ileum		2
- terminal ileum + colon		6
- colon only		6
**Endoscopic classification^1 ^(n)**		
- mild inflammation	15	4
- moderate inflammation	27	8
- severe inflammation	7	2
**Histopathologic examination (n)**		
- mild disease activity	20	1
- moderate disease activity	23	8
- severe disease activity	6	1
**Anti-inflammatory therapy [AIT, (n)]^2^**		
- no AIT	14	1
- AIT	35	13
*substance class*		
- 5-ASA	31	6
- corticosteroids	8	8
- immunomodulators	3	3

^1 ^Macroscopic classification of the degree of inflammation was adjusted according to the Mayo endoscopic subscore [19].

^2 ^Some patients received different substance classes, therefore the summation of patients in the category "substance class" exceeds the number of patients in the category "AIT". 5-ASA: 5-amino salicylic acid and its derivatives, immunomodulators: azathioprine and infliximab.

### Detection of MAP in IBD patients and controls

None of the MGIT samples were positive for MAP in the cultural investigation after 12 and 52 weeks of culture.

A total of 63 IBD patients and 21 controls were analysed for the presence of MAP specific DNA in colonic mucosa. To exclude locoregional differences in MAP infection, IBD patients from Germany (n = 21) and Norway (n = 42) were recruited. Despite a more frequent detection of MAP DNA in UC patients (20%) and controls (33%) compared to patients with CD (7%) in the total patient cohort, differences in MAP frequency between these three groups were not statistically significant (CD vs. controls: p = 0.1078, UC vs. controls: p = 0.3615, CD vs. UC: p = 0.4298).

Subgroup analyses considering the German and Norwegian patient cohort separately revealed likewise no significant differences in MAP frequency between patients with CD and UC and controls (Norway: CD vs. controls: p = 0.2945; UC vs. controls: p = 0.1885; CD vs. UC: p = 1.00 and Germany: CD vs. controls: 0.2216; UC vs. controls: p = 1.00; CD vs. UC: 0.3108). Although the MAP detection rate was generally higher in Germany (29%) than in Norway (14%), these differences did not reach the level of statistical significance (p = 0.1828). MAP prevalence in the different cohorts is summarized in Table 4.

**Table 4:** Frequency MAP DNA detection in IBD patients and controls

Patient cohort (n)	MAP positive (n)	MAP negative (n)
**Control cohort **(n = 21)	7 (33%)	14 (67%)
**IBD patients Germany**		
-CD (n = 10)	1 (10%)	9 (90%)
-UC (11)	4 (36%)	7 (64%)
**IBD patients Norway**		
-CD (n = 4)	0 (0%)	4 (100%)
-UC (n = 38)	6 (16%)	32 (84)

**IBD patients Norway + Germany**		
-CD (n = 14)	1 (7%)	13 (93%)
-UC (n = 49)	10 (20%)	39 (80%)

**Figure 1:**
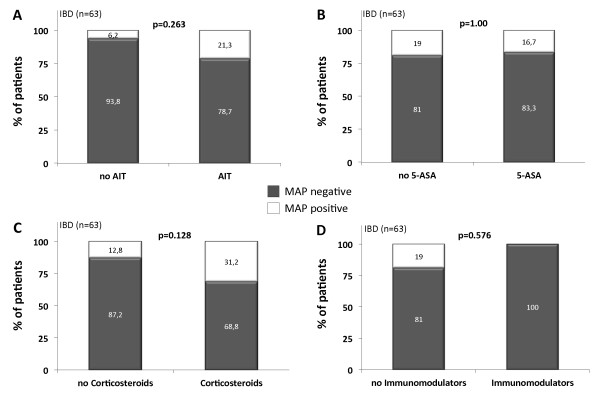
**Frequency of intestinal MAP DNA detection in subgroups of IBD patients (n = 63)**. Comparisons were made using Fisher's exact test. For visualization, the number of patients in each subgroup was converted to 100% and the percentage of MAP positive and negative patients in each subgroup is shown, respectively. Frequency of MAP specific DNA was not altered between IBD patients with anti-inflammatory therapy (AIT) and those without (A). Similarly, there were no differences in MAP frequency between IBD patients treated with 5-amino salicylic acid (5-ASA) and its derivatives (B) or immunomodulators (azathioprine and infliximab, D) and those without the respective agents. Although not statistically significant, MAP DNA was more frequently found in IBD patients treated with corticosteroids compared to those without (C).

**Figure 2:**
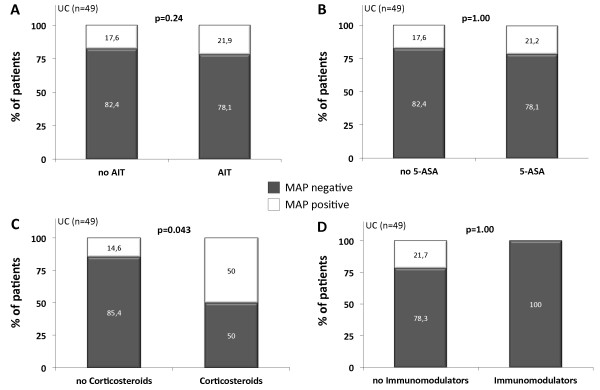
**Frequency of intestinal MAP DNA detection in subgroups of UC patients (n = 49)**. Comparisons were made using Fisher's exact test. For visualization, the number of patients in each subgroup was converted to 100% and the percentage of MAP positive and negative patients in each subgroup is shown, respectively. UC patients treated with corticosteroids had a significantly higher frequency of MAP detection than UC patients receiving no corticosteroids (C) whereas the detection of MAP DNA was not altered between IBD patients with anti-inflammatory therapy (AIT) and those without (A). Frequency of MAP specific DNA was not changed between UC patients treated with 5-amino salicylic acid (5-ASA) and its derivatives (B) or immunomodulators (azathioprine and infliximab, D) compared to those without the respective agents.

We next compared whether the frequency of MAP detection in the IBD and UC patient cohort differed depending on the applied anti-inflammatory therapy (AIT). Comparing IBD and UC patients receiving any kind of AIT to those patients without AIT, no statistical significances in MAP frequency were detected (Figure 1, 2). Similarly, no significant differences in MAP DNA prevalence were observed when patients treated with 5-amino salicylic acid (5-ASA) and its derivatives or immunomodulators (azathioprine and infliximab) were compared to those patients without the respective agents. When comparing patients stratified to the treatment with corticosteroids, MAP DNA was significantly more frequently obtained in UC patients treated with corticosteroids compared to those without corticosteroids in their therapeutic regime (p = 0.043, Figure 2c). However, although not statistically significant, a higher frequency of MAP detection in the corticosteroid group was observed in the total IBD population as well (Figure 1c).

### MMP expression in UC patients with and without presence of MAP DNA

For MMP analyses, all determined mRNA values were related to the respective content of 18S rRNA which served as a housekeeping gene. In order to account for interindividual differences in the MMP expression in the colon, MMP expression in diseased colonic mucosa was referred to the respective expression in corresponding healthy mucosa of the same individual.

Gene expression of MMP-1, MMP-2, MMP-7, MMP-9, MMP-13 and MMP-19 was unchanged between UC patients with presence of MAP DNA and those without (Figures 3, 4, 5, 6, 7, 8). Mucosal mRNA expression of MMP-28 and TNF-α did not show any dependency on the presence of MAP DNA either (Figures 9 and 10).

**Figure 3:**
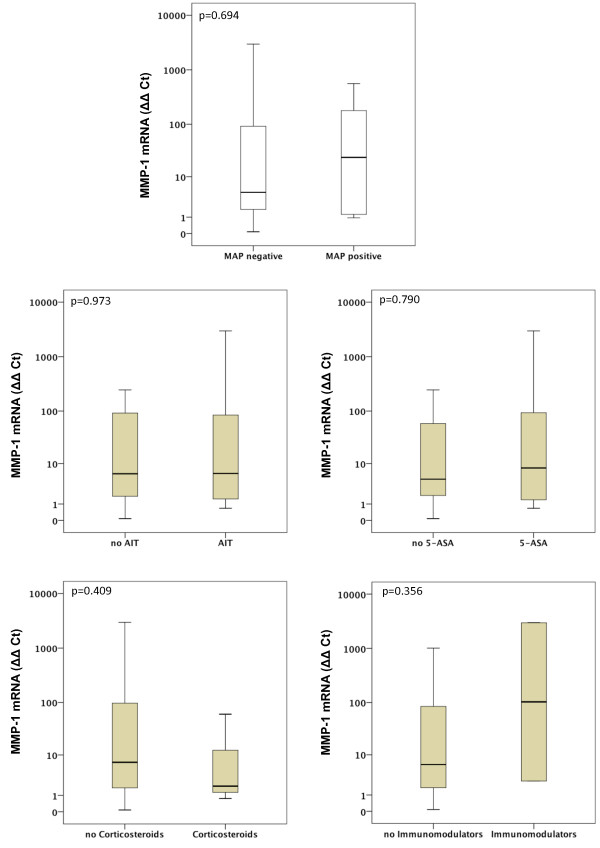
**Expression of MMP-1 in UC patients (n = 49)**. mRNA results were determined by RT-PCR. MMP-1 gene expression was not significantly different in UC patients with intestinal MAP detection and those without (p = 0.694) and did not differ between patients receiving an anti-inflammatory therapy (AIT) and those without AIT (p = 0.973). Further stratification into subgroups of patients receiving 5-amino salicylic acid (5-ASA) and its derivatives, corticosteroids or immunomodulators (azathioprine and infliximab) revealed no significant differences in MMP-1 expression between patients treated with the respective substance and those not receiving the respective agent. Note, that some patients received more than one substance class and therefore appear in more than one subgroup.

**Figure 4:**
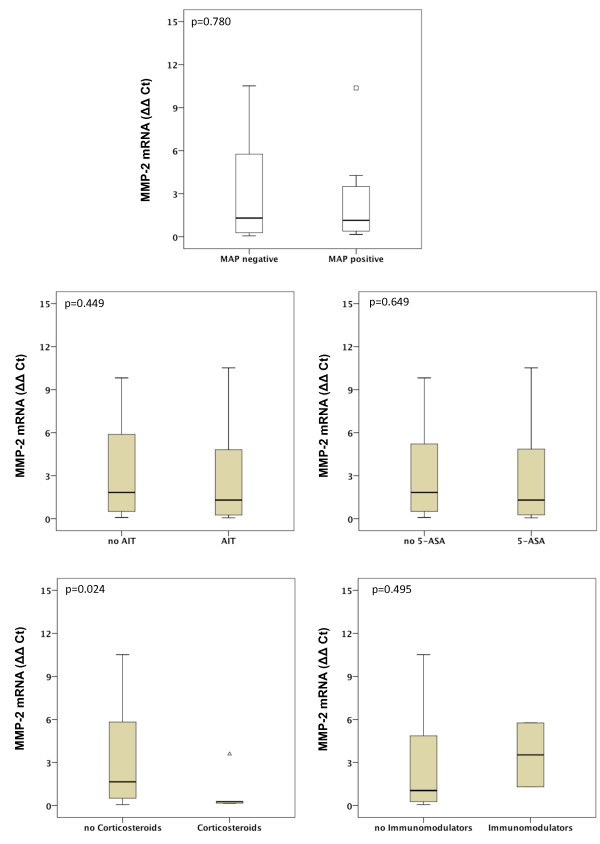
**Expression of MMP-2 in UC patients (n = 49)**. mRNA results were determined by RT-PCR. MMP-2 gene expression was not significantly different in UC patients with intestinal MAP detection and those without (p = 0.780) and did not differ between patients receiving an anti-inflammatory therapy (AIT) and those without AIT (p = 0.449). Further stratification of the UC cohort according to the different therapeutic agents revealed that MMP-2 mRNA is significantly decreased by factor 6.1 in UC patients treated with corticosteroids compared to those with no corticosteroids in their therapeutic regime (p = 0.024). No differences in MMP-2 expression were observed when patients receiving 5-amino salicylic acid (5-ASA) and its derivatives or immunomodulators (azathioprine and infliximab) were compared to patients not treated with the respective agent. Note, that some patients received more than one substance class and therefore appear in more than one subgroup.

**Figure 5:**
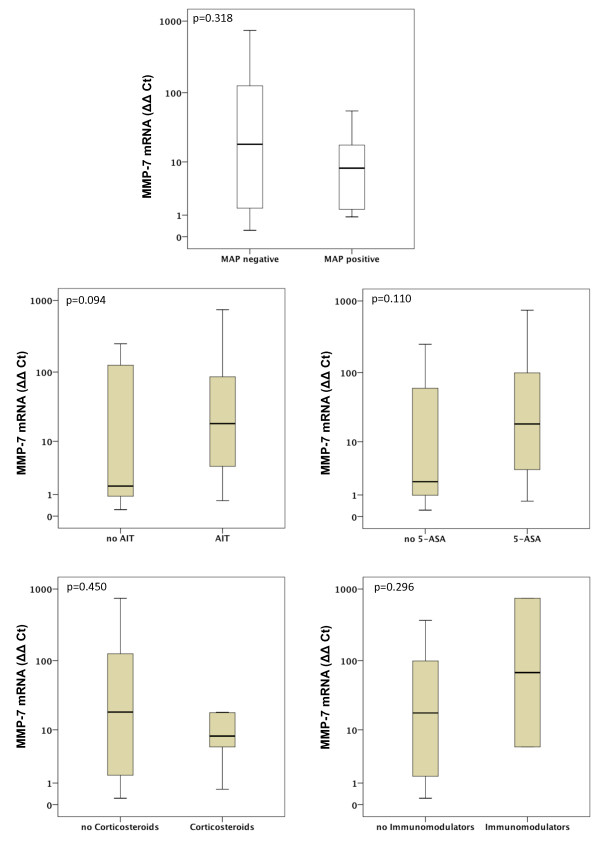
**Expression of MMP-7 in UC patients (n = 49)**. mRNA results were determined by RT-PCR. MMP-7 gene expression was not significantly different in UC patients with intestinal MAP detection and those without (p = 0.318) and did not differ between patients receiving an anti-inflammatory therapy (AIT) and those without AIT (p = 0.094). Further stratification into subgroups of patients receiving 5-amino salicylic acid (5-ASA) and its derivatives, corticosteroids or immunomodulators (azathioprine and infliximab) revealed no significant differences in MMP-7 expression between patients treated with the respective substance and those not receiving the respective agent. Note, that some patients received more than one substance class and therefore appear in more than one subgroup.

**Figure 6:**
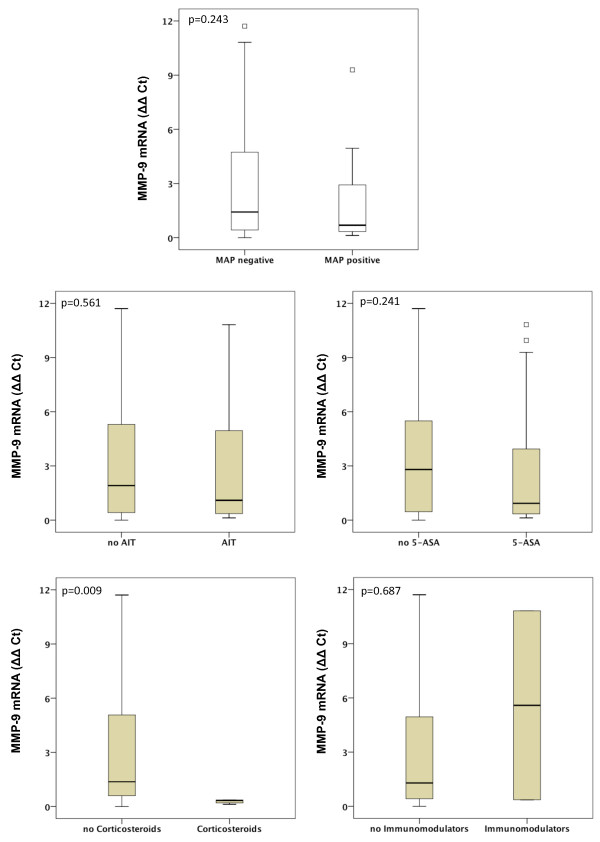
**Expression of MMP-9 in UC patients (n = 49)**. mRNA results were determined by RT-PCR. MMP-9 gene expression was not significantly different in UC patients with intestinal MAP detection and those without (p = 0.243) and did not differ between patients receiving an anti-inflammatory therapy (AIT) and those without AIT (p = 0.561). Further stratification of the UC cohort according to the different therapeutic agents revealed that MMP-9 mRNA is significantly decreased by factor 4.2 in UC patients treated with corticosteroids compared to those with no corticosteroids in their therapeutic regime (p = 0.009). No differences in MMP-9 expression were observed when patients receiving 5-amino salicylic acid (5-ASA) and its derivatives or immunomodulators (azathioprine and infliximab) were compared to patients not treated with the respective agent. Note, that some patients received more than one substance class and therefore appear in more than one subgroup.

**Figure 7:**
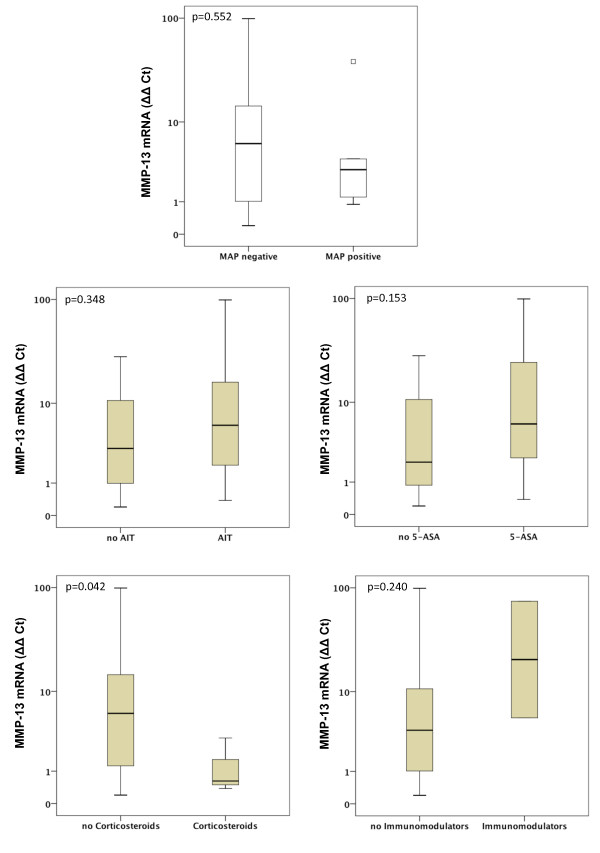
**Expression of MMP-13 in UC patients (n = 49)**. mRNA results were determined by RT-PCR. MMP-13 gene expression was not significantly different in UC patients with intestinal MAP detection and those without (p = 0.552) and did not differ between patients receiving an anti-inflammatory therapy (AIT) and those without AIT (p = 0.348). Further stratification of the UC cohort according to the different therapeutic agents revealed that MMP-13 mRNA is significantly decreased by factor 9.5 in UC patients treated with corticosteroids compared to those with no corticosteroids in their therapeutic regime (p = 0.042). No differences in MMP-13 expression were observed when patients receiving 5-amino salicylic acid (5-ASA) and its derivatives or immunomodulators (azathioprine and infliximab) were compared to patients not treated with the respective agent. Note, that some patients received more than one substance class and therefore appear in more than one subgroup.

**Figure 8:**
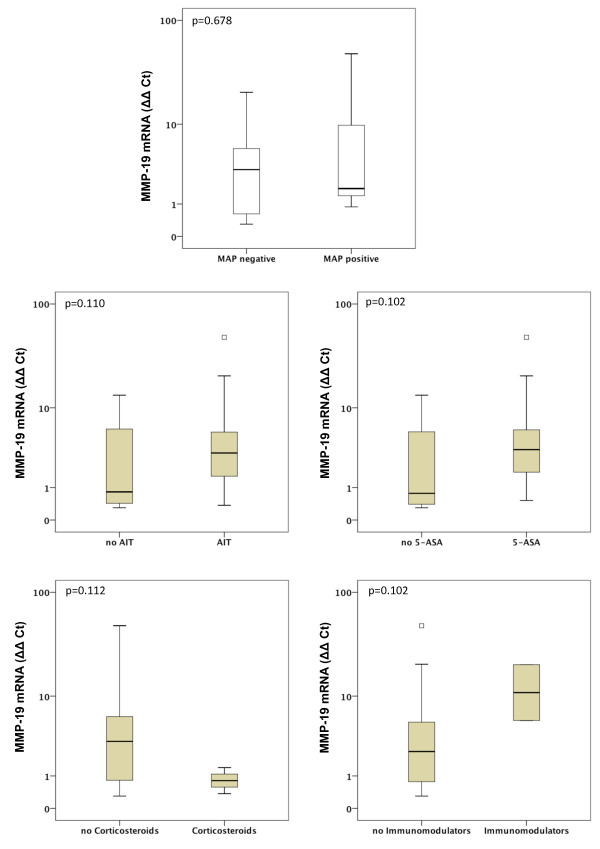
**Expression of MMP-19 in UC patients (n = 49)**. mRNA results were determined by RT-PCR. MMP-19 gene expression was not significantly different in UC patients with intestinal MAP detection and those without (p = 0.678) and did not differ between patients receiving an anti-inflammatory therapy (AIT) and those without AIT (p = 0.110). Further stratification into subgroups of patients receiving 5-amino salicylic acid (5-ASA) and its derivatives, corticosteroids or immunomodulators (azathioprine and infliximab) revealed no significant differences in MMP-19 expression between patients treated with the respective substance and those not receiving the respective agent. Note, that some patients received more than one substance class and therefore appear in more than one subgroup.

**Figure 9:**
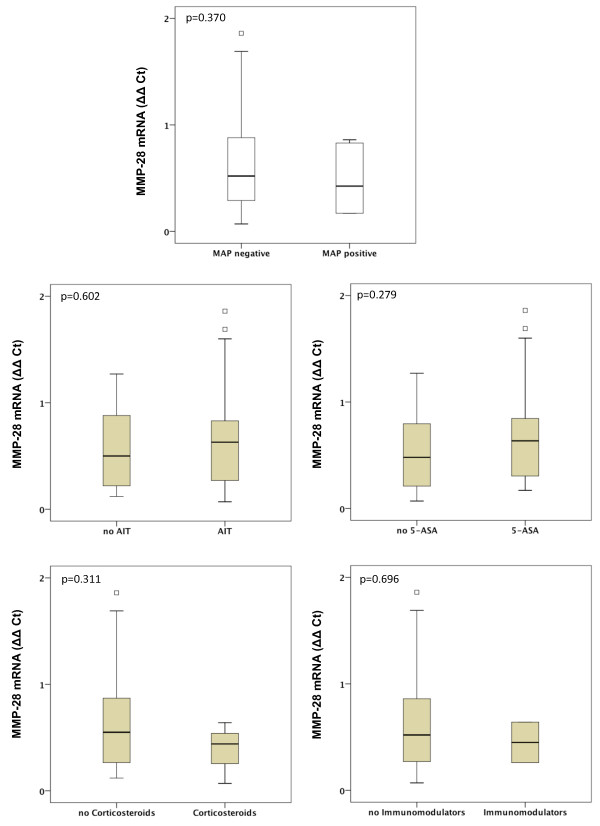
**Expression of MMP-28 in UC patients (n = 49)**. mRNA results were determined by RT-PCR. MMP-28 gene expression was not significantly different in UC patients with intestinal MAP detection and those without (p = 0.370) and did not differ between patients receiving an anti-inflammatory therapy (AIT) and those without AIT (p = 0.602). Further stratification into subgroups of patients receiving 5-amino salicylic acid (5-ASA) and its derivatives, corticosteroids or immunomodulators (azathioprine and infliximab) revealed no significant differences in MMP-28 expression between patients treated with the respective substance and those not receiving the respective agent. Note, that some patients received more than one substance class and therefore appear in more than one subgroup.

**Figure 10:**
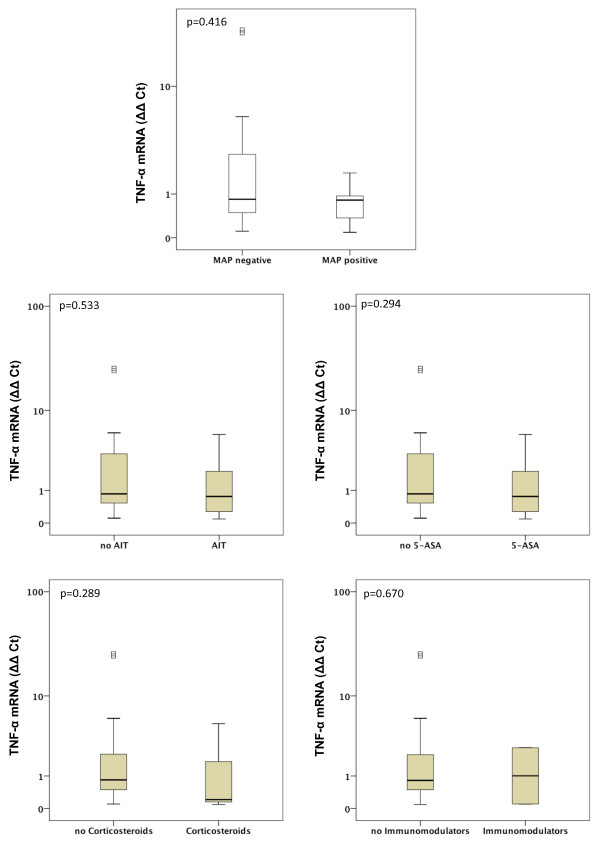
**Expression of TNF-α in UC patients (n = 49)**. mRNA results were determined by RT-PCR. TNF-α gene expression was not significantly different in UC patients with intestinal MAP detection and those without (p = 0.416) and did not differ between patients receiving an anti-inflammatory therapy (AIT) and those without AIT (p = 0.533). Further stratification into subgroups of patients receiving 5-amino salicylic acid (5-ASA) and its derivatives, corticosteroids or immunomodulators (azathioprine and infliximab) revealed no significant differences in TNF-α expression between patients treated with the respective substance and those not receiving the respective agent. Note, that some patients received more than one substance class and therefore appear in more than one subgroup.

As MAP DNA was detected in only one CD patient, we were unable to analyse MMP expression with regard to MAP status in the CD patient cohort. Since endoscopic evaluation of screening colonoscopy did not reveal any signs of intestinal inflammation, MMP regulation in inflamed mucosa expressed as a multiple of basal expression in healthy mucosa could not be performed in the control cohort either.

### Effects of anti-inflammatory agents on MMP and TNF-α expression

To analyse a potential effect of the applied anti-inflammatory therapy on intestinal MMP expression, we further calculated MMP and TNF-α expression in UC patients stratified by the different therapeutic modalities. Gene expression of MMP-1, -2, -7, -9, -13, -19, -28 and TNF-α was unchanged between patients with and without AIT, between patients stratified to 5-ASA and its derivatives and between patients with and without immunomodulators. However, median levels of MMP-2, MMP-9, and MMP-13 were significantly decreased in UC patients treated with corticosteroids compared to those with no corticosteroids in their therapeutic regime (MMP-2: 6.1-fold, p = 0.024; MMP-9: 4.2-fold, p = 0.009; MMP-13: 9.5-fold, p = 0.042, Figures 3, 4, 5, 6, 7, 8, 9, 10).

Based on this finding, we excluded steroid-treated patients in a further analysis and re-calculated the MMP and TNF-α expression in MAP positive and MAP negative IBD patients. However, despite the non-consideration of steroid treated patients, expression of MMP-1, -2, -7, -9, -13, -19, -28 and TNF-α remained unchanged between MAP positive and negative IBD patients (Additional Files 1, 2, 3, 4).

## Discussion

The histopathological and clinical similarities between human chronic granulomatous enteritis, intestinal tuberculosis, and animal paratuberculosis [3,23] has led to the suspicion that *Mycobacterium avium *subspecies *paratuberculosis *is an etiologic factor for the development of Crohn's disease. MAP is fastidious and slow-growing and can therefore only rarely be detected by conventional microbiological techniques [1]. Due to the difficulties in growing MAP *in vitro*, molecular and serological methods have been developed. Nevertheless, the molecular detection of MAP DNA has also frequently been hampered by insufficient specificity of the selected primers and probes, inadequate DNA extraction methods resulting in insufficient sensitivity, and possible contamination as a consequence of the PCR techniques used [24]. In the current study, we therefore selected a nested PCR system and a real time-PCR method instead of conventional PCR sets, which ensured a sensitive and specific approach. The nested PCR used was developed by Bull et al. [21] and is based on the widely used MAP reference marker IS*900*. The oligonucleotide primer sequences were specifically adapted to show no cross reactions with the previously described IS900-like elements [25,26]. The positive IS*900 *PCR products detected were sequenced and corresponded to MAP IS*900 *but not IS*900*-like sequences. In addition, a triplex real-time PCR assay combining the MAP-specific gene sequences F57, IS*Mav2*, and an internal amplification control for diagnostic quality assurance was applied. The real time-PCR method was carefully validated by using more than 200 references and field strains with different kinds of sample preparation techniques on artificially contaminated and naturally infected samples.

Based on 2 different PCR methods utilized in this study, false-positive results due to an unspecific binding of oligonucleotide primer pairs are therefore highly improbable. The risk of cross-contamination between the samples was also reduced by using sample preparation negative controls. The use of an IAC has been shown to be mandatory for diagnostic PCR applications to exclude false negative results [27]. The included IAC of the real-time PCR assay revealed that none of the real-time PCR reactions was inhibited.

Using this specific and sensitive approach, we explored the detection rate of MAP DNA in colonic biopsies of German and Norwegian patients with IBD and control patients without intestinal inflammation. Within this study, we found no significant differences in MAP DNA detection frequency between patients with UC and CD and controls.

Regarding the literature, reports about the association of MAP and CD are controversial. So far, a number of studies have reported more frequent detection of MAP DNA in CD patients compared with controls using PCR assays [21,28,29]. Other authors, in contrast, found no association between CD and the detection of MAP DNA [30-35]. Nevertheless, comparisons between the studies are difficult to make due to heterogeneity in the analysed specimens that vary from fresh [35,36] to embedded tissue [30,31] and blood [28,29,32-34]. Furthermore, different MAP detection methods are used across the studies including MAP DNA [21,28,30,31,33-35], MAP culture [29,32,34], both DNA and culture [34], specific antibodies against MAP epitopes [28] and immunocytochemistry [31]. Although recent meta-analyses were able to confirm the association of MAP infection and CD [4,6], the pathogenic significance of this association remains unclear. Within our study, we detected MAP DNA in only a low percentage of CD patients. As a limiting factor, our cohort of patients with CD was relatively small. *Post hoc *power analysis of the CD population against an alpha level of 0.05 estimated a two-tailed statistical power of 0.135. Consequently, appropriate conclusions about an etiologic involvement of MAP in the pathogenesis of CD cannot be drawn from the results of our study.

During the initial phase of our study, we detected MAP DNA in an unexpected high percentage of German UC patients. To exclude a locoregional influence or epiphenomenon, we aimed to confirm this result in a distinct UC population and therefore expanded our analyses to a cohort of Norwegian UC patients. In this cohort, we likewise observed a high frequency of MAP DNA. Data on the frequency of MAP detection in UC in the literature are contradictory as well. In general, MAP is more frequently detected in CD than in UC according to recent meta-analyses [4,6]. Nevertheless, several studies detected MAP infection in a high percentage of UC patients as well [7,28,37-40].

As a further remarkable result, we detected MAP DNA in intact colonic mucosa in a high percentage of German control patients (33%). As both, the Norwegian and German patient population represent an urban population with a comparable demographic and epidemiologic structure and a similar BCG vaccination policy in Germany and Norway, we did not assess the colonic MAP prevalence in healthy Norwegians. In conjunction with our results, previous studies have made similar observations by serologically detecting MAP DNA or specific MAP antibodies in a high percentage of healthy control patients [37,39-41]. In this context, the following aspects are important to note: MAP has been cultured from chlorinated potable municipal water [42]. Mycobacteria are at least two orders of magnitude more resistant to chlorine purification than *Escherichia coli *and MAP survives higher concentrations of chlorine than the 1.1 parts per million routinely achieved with first-use municipal water in the USA [43]. Furthermore, MAP has been cultured from pasteurized milk in the USA and Europe [44-46]. Considering these facts, it has been speculated that the frequent detection of MAP in healthy controls is a result of the wide distribution of MAP and its presence in the food-chain. Nevertheless, the biological significance of this finding remains unclear as the detection of MAP DNA might well come from live bacteria but also might merely represent scattered debris from killed bacteria.

Anti-inflammatory agents such as 5-ASA and its derivatives, corticosteroids and immunomodulators are established therapeutic agents for the treatment of IBD. Most interestingly, it has been shown that 5-ASA, thalidomide, methotrexate, 6-mercaptopurine and azathioprine inhibit MAP *in vitro *in a dose dependent manner [47-50]. Further, recent evidence suggests that 5-ASA decreases the prevalence of MAP DNA from the blood of IBD patients whereas other agents such as 6-mercaptopurin, methotrexate and tacrolimus are associated with clearance of MAP DNA from the blood [39]. To analyse a potential effect of the applied anti-inflammatory therapy on the MAP DNA prevalence in intestinal tissue, we performed subgroup analyses according to the different therapeutic agents in our IBD cohort. However, we were not able to detect significant differences in the MAP DNA prevalence between IBD and UC patients with and without anti-inflammatory therapy and between those patients receiving 5-ASA or immunomodulators compared to patients without the respective agents. Based on the unrefuted data of the anti-MAP activities of IBD medication mentioned above, we assume that the most likely explanation for the lack of association between 5-ASA and immunomodulators and MAP detection rates within our study, apart from the different specimens analysed (tissue vs. blood), is the relative small sample size in our study. Nevertheless, our results provide evidence that UC patients treated with corticosteroids have a higher frequency of intestinal MAP DNA than those not treated with corticosteroids. This result is in accordance with observations from Naser et al. who cultured MAP from blood in 60% (3 of 5) of IBD patients treated with steroids [29]. Although a larger study by Juste and co-workers did not detect a significant increase in MAP prevalence in patients receiving steroids, the authors suggested that the lack of increased MAP DNA frequency in steroid treated IBD patients in their study might be attributable to frequent co-administration of anti-MAP agents in this group [39]. Furthermore, the authors concluded that caution suggests that concomitant anti-MAP agents should always be used when steroids are administered [39], a position that is strengthened by the results of our study.

To further evaluate a potential contribution of MAP infection to disease manifestation in UC patients, we analysed the expression of Matrix Metalloproteinases with regard to the presence or absence of intestinal MAP DNA. Members of the MMP family have been shown to be upregulated in peripheral blood mononuclear cells from cattle infected with Johne's disease after stimulation with MAP [16,17]. Furthermore, murine *in vitro *and *in vivo *studies provide evidence that upregulation of certain MMPs such as MMP-2 and MMP-9 plays a pathogenic role in infections caused by pathogenic mycobacteria [18]. Most interestingly, enhanced MMP activity after mycobacterial infection was substantially reduced upon treatment with anti-TNFα antibodies [18], a treatment strategy which has been proven to be highly beneficial in human IBD [51,52]. On the other hand, *in vitro *data from Janagama and co-workers demonstrate that MAP strains from diverse hosts differ in their ability to survive intracellularly and that strains with higher intracellular persistence had decreased expression of different cytokines and MMP-3 [53]. Thus, an anti-inflammatory and anti-invasive milieu might facilitate their persistence and survival within macrophages [53].

On the basis of this evidence, we aimed to analyse the mucosal MMP expression in IBD patients with and without intestinal MAP DNA. Unfortunately, we had to restrict the analysis to the UC patient cohort, as MAP DNA was detected in only one CD patient. Using this approach we focused on MMP gene expression as several studies have shown that expression of MMPs is mainly transcriptional regulated with close correlation between MMP protein concentrations and mRNA expression [54-57]. We analysed a broad spectrum of MMPs including MMP-1, MMP-7 and MMP-13, which have been proven to be major proteases in the pathogenesis of IBD associated mucosal ulcerations [13-15,58,59], and MMP-2 and MMP-9, which have been shown to be upregulated upon mycobacterial infection [18]. Furthermore, we determined the expression of MMP-28, the most recent member of the MMP family which is expressed in intact colonic epithelium rather than in UC affected mucosa and therefore is associated with epithelial integrity [14,60]. Our results provide evidence that neither expression of MMP-1, -2, -7, -9, -13, -19 nor of MMP-28 is altered in UC patients with presence of intestinal MAP DNA compared to those without MAP detection. TNF-α, which regulates MMP-2 and MMP-9 and is part of the cytokine response towards MAP in CD patients [10,11,61,62], was unchanged between UC patients with intestinal MAP DNA detection and those without. Subgroup analyses based on different anti-inflammatory agents applied in our IBD cohort revealed significantly decreased MMP-2, MMP-9, and MMP-13 gene transcripts in UC patients treated with corticosteroids compared to those with no corticosteroids in their therapeutic regime (MMP-2: 6.1-fold, p = 0.024; MMP-9: 4.2-fold, p = 0.009; MMP-13: 9.5-fold, p = 0.042). However, when we excluded steroid-treated patients in a further analysis and re-calculated the MMP and TNF-α expression in MAP positive and MAP negative IBD patients, no significant differences were observed between MAP positive and MAP negative patients (Additional Files 1, 2, 3, 4).

To further elucidate the pathogenic potential of MAP for the development of IBD and its significance in healthy individuals, it seems inevitable to transfer the results of the observed association of MAP and IBD to more functional studies in the future. To understand different susceptibilities towards MAP infections, it will be of great value to determine the presence or absence of certain genetic IBD risk loci in CD and UC patients. Among others, NOD2 and Nramp1 might be of particular interest in the context of MAP infection: approximately 30% of European ancestry have one of three NOD2 polymorphisms and homozygosity for one polymorphism confers an increased risk for CD by factor 11 to 27 [2]. Interestingly, embryonic kidney cells transfected with NOD2 are able to recognize MAP and respond in a dose dependent manner [63]. Defects and mutations in Nrmap (natural-resistance-associated macrophage protein 1) are associated with increased susceptibility to mycobacterial infections in humans and animals and in both, UC and CD patients have a frequency of Nramp1 promoter polymorphisms than healthy controls [64]. Another potential approach might be the use murine models with knock-down of certain IBD susceptibility loci and subsequent controlled infection with MAP strains and other mycobacteria.

## Conclusions

Our study provides evidence that MAP can frequently be detected in intestinal mucosa of patients suffering from ulcerative colitis as well as from control patients without intestinal inflammation. This result might well be attributable to the wide environmental distribution of MAP and its presence in the food-chain. Furthermore, we are able to show that corticosteroids are associated with increased detection of intestinal MAP DNA and decreased expression of certain MMPs. The results of this study provide further evidence that the presence of intestinal MAP specific DNA is not associated with altered MMP expression in ulcerative colitis *in vivo*. Hence, the biological significance of MAP and its contribution to the pathogenesis of IBD remains elusive.

## Competing interests

TR has no competing interests to disclose. MR has no competing interests to disclose. SB has no competing interests to disclose. AR has no competing interests to disclose. TB has no competing interests to disclose. ÖA has no competing interests to disclose. AA has no competing interests to disclose. JMH has no competing interests to disclose. RG has no competing interests to disclose. MB has no competing interests to disclose. ER has no competing interests to disclose.

## Authors' contributions

TR participated in the collection the biopsy samples, determined the MMP expression, analysed the data and drafted the manuscript (together with ER). MR, SB and AR participated in study design, collection of the biopsy samples and MMP analyses. ÖA, AA and MB performed the MAP detection and critically revised the manuscript. JMH participated in the collection of the biopsy samples and MMP analyses. TB and RG provided important intellectual content and participated in study design. ER designed the study, analysed the data and drafted the manuscript (together with TR). All authors read and approved the final manuscript.

## Pre-publication history

The pre-publication history for this paper can be accessed here:

http://www.biomedcentral.com/1471-230X/11/34/prepub

## Supplementary Material

Additional file 1 **Expression of MMP-1 and MMP-2 in UC patients without corticosteroids with respect to the presence of MAP DNA (n = 41)**. mRNA results were determined by RT-PCR. MMP-1 and MMP-2 gene expression was not significantly different in steroidfree UC patients with intestinal MAP detection compared to those without. Click here for file

Additional file 2 **Expression of MMP-7 and MMP-9 in UC patients without corticosteroids with respect to the presence of MAP DNA (n = 41)**. mRNA results were determined by RT-PCR. MMP-7 and MMP-9 gene expression was not significantly different in steroidfree UC patients with intestinal MAP detection compared to those without. Click here for file

Additional file 3 **Expression of MMP-13 and MMP-19 in UC patients without corticosteroids with respect to the presence of MAP DNA (n = 41)**. mRNA results were determined by RT-PCR. MMP-13 and MMP-19 gene expression was not significantly different in steroidfree UC patients with intestinal MAP detection compared to those without. Click here for file

Additional file 4 **Expression of MMP-28 and TNF-α in UC patients without corticosteroids with respect to the presence of MAP DNA (n = 41)**. mRNA results were determined by RT-PCR. MMP-28 and TNF-α gene expression was not significantly different in steroidfree UC patients with intestinal MAP detection compared to those without. Click here for file

## References

[B1] Tiwari A VanLeeuwen JA McKenna SL Keefe GP Barkema HW Johne's disease in Canada Part I: clinical symptoms, pathophysiology, diagnosis, and prevalence in dairy herds Can Vet J 2006 47 874 882 17017652 PMC1555680

[B2] Abraham C Cho JH Inflammatory bowel disease N Engl J Med 2009 361 2066 2078 10.1056/NEJMra0804647 19923578 PMC3491806

[B3] Dalziel TK Chronical intestinal enteritis Br Med J 1913 2 1068 1070

[B4] Abubakar I Myhill D Aliyu SH Hunter PR Detection of Mycobacterium avium subspecies paratuberculosis from patients with Crohn's disease using nucleic acid-based techniques: a systematic review and meta-analysis Inflamm Bowel Dis 2008 14 401 410 10.1002/ibd.20276 17886288

[B5] Behr MA Kapur V The evidence for Mycobacterium paratuberculosis in Crohn's disease Curr Opin Gastroenterol 2008 24 17 21 10.1097/MOG.0b013e3282f1dcc4 18043227

[B6] Feller M Huwiler K Stephan R Altpeter E Shang A Furrer H Pfyffer GE Jemmi T Baumgartner A Egger M Mycobacterium avium subspecies paratuberculosis and Crohn's disease: a systematic review and meta-analysis Lancet Infect Dis 2007 7 607 613 10.1016/S1473-3099(07)70211-6 17714674

[B7] Kirkwood CD Wagner J Boniface K Vaughan J Michalski WP Catto-Smith AG Cameron DJ Bishop RF Mycobacterium avium subspecies paratuberculosis in children with early-onset Crohn's disease Inflamm Bowel Dis 2009 15 1643 1655 10.1002/ibd.20967 19462429

[B8] Chamberlin WM Naser SA Integrating theories of the etiology of Crohn's disease. On the etiology of Crohn's disease: questioning the hypotheses Med Sci Monit 2006 12 RA27 33 16449960

[B9] Mendoza JL Lana R Diaz-Rubio M Mycobacterium avium subspecies paratuberculosis and its relationship with Crohn's disease World J Gastroenterol 2009 15 417 422 10.3748/wjg.15.417 19152445 PMC2653362

[B10] Clancy R Ren Z Turton J Pang G Wettstein A Molecular evidence for Mycobacterium avium subspecies paratuberculosis (MAP) in Crohn's disease correlates with enhanced TNF-alpha secretion Dig Liver Dis 2007 39 445 451 10.1016/j.dld.2006.12.006 17317344

[B11] Sibartie S Scully P Keohane J O'Neill S O'Mahony J O'Hanlon D Kirwan WO O'Mahony L Shanahan F Mycobacterium avium subsp. Paratuberculosis (MAP) as a modifying factor in Crohn's disease Inflamm Bowel Dis 2010 16 296 304 19824071 10.1002/ibd.21052

[B12] Parks WC Wilson CL Lopez-Boado YS Matrix metalloproteinases as modulators of inflammation and innate immunity Nat Rev Immunol 2004 4 617 629 10.1038/nri1418 15286728

[B13] Rath T Roderfeld M Graf J Roeb E [Matrix metalloproteinases in inflammatory bowel disease - from basic research to clinical significance] Z Gastroenterol 2009 47 758 769 10.1055/s-0028-1109520 19662589

[B14] Rath T Roderfeld M Halwe JM Tschuschner A Roeb E Graf J Cellular sources of MMP-7, MMP-13 and MMP-28 in ulcerative colitis Scand J Gastroenterol 2010 45 1186 1196 10.3109/00365521.2010.499961 20568971

[B15] Ravi A Garg P Sitaraman SV Matrix metalloproteinases in inflammatory bowel disease: boon or a bane? Inflamm Bowel Dis 2007 13 97 107 10.1002/ibd.20011 17206645

[B16] Coussens PM Colvin CJ Rosa GJ Perez Laspiur J Elftman MD Evidence for a novel gene expression program in peripheral blood mononuclear cells from Mycobacterium avium subsp. paratuberculosis-infected cattle Infect Immun 2003 71 6487 6498 10.1128/IAI.71.11.6487-6498.2003 14573671 PMC219592

[B17] Coussens PM Pudrith CB Skovgaard K Ren X Suchyta SP Stabel JR Heegaard PM Johne's disease in cattle is associated with enhanced expression of genes encoding IL-5, GATA-3, tissue inhibitors of matrix metalloproteinases 1 and 2, and factors promoting apoptosis in peripheral blood mononuclear cells Vet Immunol Immunopathol 2005 105 221 234 10.1016/j.vetimm.2005.02.009 15808302

[B18] Quiding-Jarbrink M Smith DA Bancroft GJ Production of matrix metalloproteinases in response to mycobacterial infection Infect Immun 2001 69 5661 5670 10.1128/IAI.69.9.5661-5670.2001 11500442 PMC98682

[B19] Schroeder KW Tremaine WJ Ilstrup DM Coated oral 5-aminosalicylic acid therapy for mildly to moderately active ulcerative colitis. A randomized study N Engl J Med 1987 317 1625 1629 10.1056/NEJM198712243172603 3317057

[B20] Schonenbrucher H Abdulmawjood A Failing K Bulte M New triplex real-time PCR assay for detection of Mycobacterium avium subsp. paratuberculosis in bovine feces Appl Environ Microbiol 2008 74 2751 2758 10.1128/AEM.02534-07 18326682 PMC2394907

[B21] Bull TJ McMinn EJ Sidi-Boumedine K Skull A Durkin D Neild P Rhodes G Pickup R Hermon-Taylor J Detection and verification of Mycobacterium avium subsp. paratuberculosis in fresh ileocolonic mucosal biopsy specimens from individuals with and without Crohn's disease J Clin Microbiol 2003 41 2915 2923 10.1128/JCM.41.7.2915-2923.2003 12843021 PMC165291

[B22] Pfaffl MW A new mathematical model for relative quantification in real-time RT-PCR Nucleic Acids Res 2001 29 e45 10.1093/nar/29.9.e45 11328886 PMC55695

[B23] Chacon O Bermudez LE Barletta RG Johne's disease, inflammatory bowel disease, and Mycobacterium paratuberculosis Annu Rev Microbiol 2004 58 329 363 10.1146/annurev.micro.58.030603.123726 15487941

[B24] Harris NB Barletta RG Mycobacterium avium subsp. paratuberculosis in Veterinary Medicine Clin Microbiol Rev 2001 14 489 512 10.1128/CMR.14.3.489-512.2001 11432810 PMC88986

[B25] Cousins DV Whittington R Marsh I Masters A Evans RJ Kluver P Mycobacteria distenct from Mycobacterium avium subsp. paratuberculosis isolated from the faeces of ruminants possess IS900-like sequences detectable IS900 polymerase chain reaction: implications for diagnosis Mol Cell Probes 1999 13 431 442 10.1006/mcpr.1999.0275 10657148

[B26] Englund S Bolske G Johansson KE An IS900-like sequence found in a Mycobacterium sp. other than Mycobacterium avium subsp. paratuberculosis FEMS Microbiol Lett 2002 209 267 271 10.1111/j.1574-6968.2002.tb11142.x 12007816

[B27] Hoorfar J Cook N Malorny B Wagner M De Medici D Abdulmawjood A Fach P Diagnostic PCR: making internal amplification control mandatory Lett Appl Microbiol 2004 38 79 80 10.1046/j.1472-765X.2003.01456.x 14746535

[B28] Di Sabatino A Paccagnini D Vidali F Rosu V Biancheri P Cossu A Zanetti S Corazza GR Sechi LA Detection of Mycobacterium avium subsp. paratuberculosis (MAP)-specific IS900 DNA and antibodies against MAP peptides and lysate in the blood of Crohn's disease patients Inflamm Bowel Dis 2010 10.1002/ibd.21461 20815038

[B29] Naser SA Ghobrial G Romero C Valentine JF Culture of Mycobacterium avium subspecies paratuberculosis from the blood of patients with Crohn's disease Lancet 2004 364 1039 1044 10.1016/S0140-6736(04)17058-X 15380962

[B30] Baksh FK Finkelstein SD Ariyanayagam-Baksh SM Swalsky PA Klein EC Dunn JC Absence of Mycobacterium avium subsp. paratuberculosis in the microdissected granulomas of Crohn's disease Mod Pathol 2004 17 1289 1294 10.1038/modpathol.3800184 15154014

[B31] Ellingson JL Cheville JC Brees D Miller JM Cheville NF Absence of Mycobacterium avium subspecies paratuberculosis components from Crohn's disease intestinal biopsy tissues Clin Med Res 2003 1 217 226 10.3121/cmr.1.3.217 15931311 PMC1069047

[B32] Freeman H Noble M Lack of evidence for Mycobacterium avium subspecies paratuberculosis in Crohn's disease Inflamm Bowel Dis 2005 11 782 783 10.1097/01.MIB.0000179317.27132.24 16043998

[B33] Lozano-Leon A Barreiro-de Acosta M Dominguez-Munoz JE Absence of Mycobacterium avium subspecies paratuberculosis in Crohn's disease patients Inflamm Bowel Dis 2006 12 1190 1192 10.1097/01.mib.0000236931.58793.22 17119397

[B34] Parrish NM Radcliff RP Brey BJ Anderson JL Clark DL Koziczkowski JJ Ko CG Goldberg ND Brinker DA Carlson RA Absence of mycobacterium avium subsp. paratuberculosis in Crohn's patients Inflamm Bowel Dis 2009 15 558 565 10.1002/ibd.20799 19058231

[B35] Sasikala M Reddy DN Pratap N Sharma SK Balkumar PR Sekaran A Banerjee R Reddy DB Absence of Mycobacterium avium ss paratuberculosis-specific IS900 sequence in intestinal biopsy tissues of Indian patients with Crohn's disease Indian J Gastroenterol 2009 28 169 174 10.1007/s12664-009-0068-2 20107965

[B36] Bull TJ Hermon-Taylor J Pavlik I El-Zaatari F Tizard M Characterization of IS900 loci in Mycobacterium avium subsp. paratuberculosis and development of multiplex PCR typing Microbiology 2000 146 Pt 9 2185 2197 10974106 10.1099/00221287-146-9-2185

[B37] Bernstein CN Blanchard JF Rawsthorne P Collins MT Population-based case control study of seroprevalence of Mycobacterium paratuberculosis in patients with Crohn's disease and ulcerative colitis J Clin Microbiol 2004 42 1129 1135 10.1128/JCM.42.3.1129-1135.2004 15004064 PMC356871

[B38] Collins MT Lisby G Moser C Chicks D Christensen S Reichelderfer M Hoiby N Harms BA Thomsen OO Skibsted U Binder V Results of multiple diagnostic tests for Mycobacterium avium subsp. paratuberculosis in patients with inflammatory bowel disease and in controls J Clin Microbiol 2000 38 4373 4381 11101567 10.1128/jcm.38.12.4373-4381.2000 PMC87608

[B39] Juste RA Elguezabal N Garrido JM Pavon A Geijo MV Sevilla I Cabriada JL Tejada A Garcia-Campos F Casado R On the prevalence of M. avium subspecies paratuberculosis DNA in the blood of healthy individuals and patients with inflammatory bowel disease PLoS One 2008 3 e2537 10.1371/journal.pone.0002537 18596984 PMC2434204

[B40] Mendoza JL San-Pedro A Culebras E Cies R Taxonera C Lana R Urcelay E de la Torre F Picazo JJ Diaz-Rubio M High prevalence of viable Mycobacterium avium subspecies paratuberculosis in Crohn's disease World J Gastroenterol 2010 16 4558 4563 10.3748/wjg.v16.i36.4558 20857526 PMC2945487

[B41] Juste RA Elguezabal N Pavon A Garrido JM Geijo M Sevilla I Cabriada JL Tejada A Garcia-Campos F Casado R Association between Mycobacterium avium subsp. paratuberculosis DNA in blood and cellular and humoral immune response in inflammatory bowel disease patients and controls Int J Infect Dis 2009 13 247 254 10.1016/j.ijid.2008.06.034 18922720

[B42] Mishina D Katsel P Brown ST Gilberts EC Greenstein RJ On the etiology of Crohn disease Proc Natl Acad Sci USA 1996 93 9816 9820 10.1073/pnas.93.18.9816 8790414 PMC38512

[B43] Whan LB Grant IR Ball HJ Scott R Rowe MT Bactericidal effect of chlorine on Mycobacterium paratuberculosis in drinking water Lett Appl Microbiol 2001 33 227 231 10.1046/j.1472-765x.2001.00987.x 11555209

[B44] Ayele WY Svastova P Roubal P Bartos M Pavlik I Mycobacterium avium subspecies paratuberculosis cultured from locally and commercially pasteurized cow's milk in the Czech Republic Appl Environ Microbiol 2005 71 1210 1214 10.1128/AEM.71.3.1210-1214.2005 15746320 PMC1065148

[B45] Ellingson JL Anderson JL Koziczkowski JJ Radcliff RP Sloan SJ Allen SE Sullivan NM Detection of viable Mycobacterium avium subsp. paratuberculosis in retail pasteurized whole milk by two culture methods and PCR J Food Prot 2005 68 966 972 15895728 10.4315/0362-028x-68.5.966

[B46] Grant IR Hitchings EI McCartney A Ferguson F Rowe MT Effect of commercial-scale high-temperature, short-time pasteurization on the viability of Mycobacterium paratuberculosis in naturally infected cows' milk Appl Environ Microbiol 2002 68 602 607 10.1128/AEM.68.2.602-607.2002 11823197 PMC126679

[B47] Greenstein RJ Su L Brown ST On the effect of thalidomide on Mycobacterium avium subspecies paratuberculosis in culture Int J Infect Dis 2009 13 e254 263 10.1016/j.ijid.2008.10.016 19303801

[B48] Greenstein RJ Su L Haroutunian V Shahidi A Brown ST On the action of methotrexate and 6-mercaptopurine on M. avium subspecies paratuberculosis PLoS One 2007 2 e161 10.1371/journal.pone.0000161 17252054 PMC1779805

[B49] Greenstein RJ Su L Shahidi A Brown ST On the action of 5-amino-salicylic acid and sulfapyridine on M. avium including subspecies paratuberculosis PLoS One 2007 2 e516 10.1371/journal.pone.0000516 17565369 PMC1885215

[B50] Shin SJ Collins MT Thiopurine drugs azathioprine and 6-mercaptopurine inhibit Mycobacterium paratuberculosis growth in vitro Antimicrob Agents Chemother 2008 52 418 426 10.1128/AAC.00678-07 18070971 PMC2224720

[B51] Rutgeerts P D'Haens G Targan S Vasiliauskas E Hanauer SB Present DH Mayer L Van Hogezand RA Braakman T DeWoody KL Efficacy and safety of retreatment with anti-tumor necrosis factor antibody (infliximab) to maintain remission in Crohn's disease Gastroenterology 1999 117 761 769 10.1016/S0016-5085(99)70332-X 10500056

[B52] Rutgeerts P Sandborn WJ Feagan BG Reinisch W Olson A Johanns J Travers S Rachmilewitz D Hanauer SB Lichtenstein GR Infliximab for induction and maintenance therapy for ulcerative colitis N Engl J Med 2005 353 2462 2476 10.1056/NEJMoa050516 16339095

[B53] Janagama HK Jeong K Kapur V Coussens P Sreevatsan S Cytokine responses of bovine macrophages to diverse clinical Mycobacterium avium subspecies paratuberculosis strains BMC Microbiol 2006 6 10 10.1186/1471-2180-6-10 16478544 PMC1382238

[B54] Graham MF Willey A Zhu YN Yager DR Sugerman HJ Diegelmann RF Corticosteroids repress the interleukin 1 beta-induced secretion of collagenase in human intestinal smooth muscle cells Gastroenterology 1997 113 1924 1929 10.1016/S0016-5085(97)70012-X 9394732

[B55] Kohn EC Jacobs W Kim YS Alessandro R Stetler-Stevenson WG Liotta LA Calcium influx modulates expression of matrix metalloproteinase-2 (72-kDa type IV collagenase, gelatinase A) J Biol Chem 1994 269 21505 21511 8063786

[B56] MacNaul KL Chartrain N Lark M Tocci MJ Hutchinson NI Discoordinate expression of stromelysin, collagenase, and tissue inhibitor of metalloproteinases-1 in rheumatoid human synovial fibroblasts. Synergistic effects of interleukin-1 and tumor necrosis factor-alpha on stromelysin expression J Biol Chem 1990 265 17238 17245 1698773

[B57] Rath T Roderfeld M Graf J Wagner S Vehr AK Dietrich C Geier A Roeb E Enhanced expression of MMP-7 and MMP-13 in inflammatory bowel disease: a precancerous potential? Inflamm Bowel Dis 2006 12 1025 1035 10.1097/01.mib.0000234133.97594.04 17075343

[B58] Arihiro S Ohtani H Hiwatashi N Torii A Sorsa T Nagura H Vascular smooth muscle cells and pericytes express MMP-1, MMP-9, TIMP-1 and type I procollagen in inflammatory bowel disease Histopathology 2001 39 50 59 10.1046/j.1365-2559.2001.01142.x 11454044

[B59] Matsuno K Adachi Y Yamamoto H Goto A Arimura Y Endo T Itoh F Imai K The expression of matrix metalloproteinase matrilysin indicates the degree of inflammation in ulcerative colitis J Gastroenterol 2003 38 348 354 10.1007/s005350300062 12743774

[B60] Bister VO Salmela MT Karjalainen-Lindsberg ML Uria J Lohi J Puolakkainen P Lopez-Otin C Saarialho-Kere U Differential expression of three matrix metalloproteinases, MMP-19, MMP-26, and MMP-28, in normal and inflamed intestine and colon cancer Dig Dis Sci 2004 49 653 661 10.1023/B:DDAS.0000026314.12474.17 15185874

[B61] Han YP Tuan TL Wu H Hughes M Garner WL TNF-alpha stimulates activation of pro-MMP2 in human skin through NF-(kappa)B mediated induction of MT1-MMP J Cell Sci 2001 114 131 139 11112697 10.1242/jcs.114.1.131 PMC2435089

[B62] Han YP Nien YD Garner WL Tumor necrosis factor-alpha-induced proteolytic activation of pro-matrix metalloproteinase-9 by human skin is controlled by down-regulating tissue inhibitor of metalloproteinase-1 and mediated by tissue-associated chymotrypsin-like proteinase J Biol Chem 2002 277 27319 27327 10.1074/jbc.M202842200 12004062 PMC2651824

[B63] Ferwerda G Kullberg BJ de Jong DJ Girardin SE Langenberg DM van Crevel R Ottenhoff TH Van der Meer JW Netea MG Mycobacterium paratuberculosis is recognized by Toll-like receptors and NOD2 J Leukoc Biol 2007 82 1011 1018 10.1189/jlb.0307147 17652449

[B64] Kojima Y Kinouchi Y Takahashi S Negoro K Hiwatashi N Shimosegawa T Inflammatory bowel disease is associated with a novel promoter polymorphism of natural resistance-associated macrophage protein 1 (NRAMP1) gene Tissue Antigens 2001 58 379 384 10.1034/j.1399-0039.2001.580606.x 11929588

