# Supplementary figures and images for: Presence of intestinal Mycobacterium avium subspecies paratuberculosis (MAP) DNA is not associated with altered MMP expression in ulcerative colitis
Source: BMC Gastroenterol. 2011 Apr 8;11:34. doi: 10.1186/1471-230X-11-34 (PMC3080338; doi:10.1186/1471-230X-11-34)

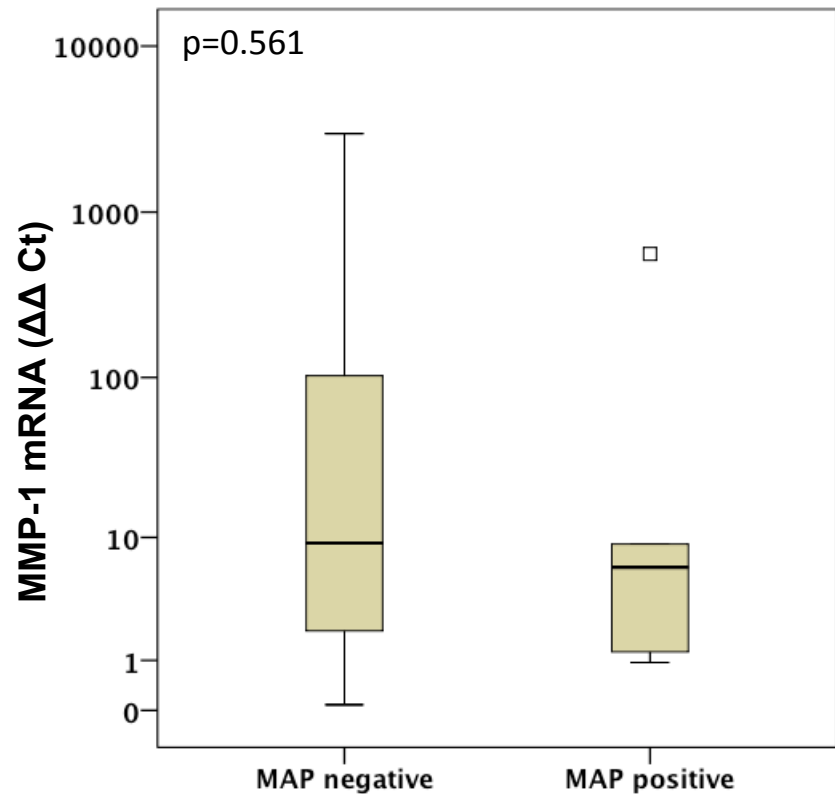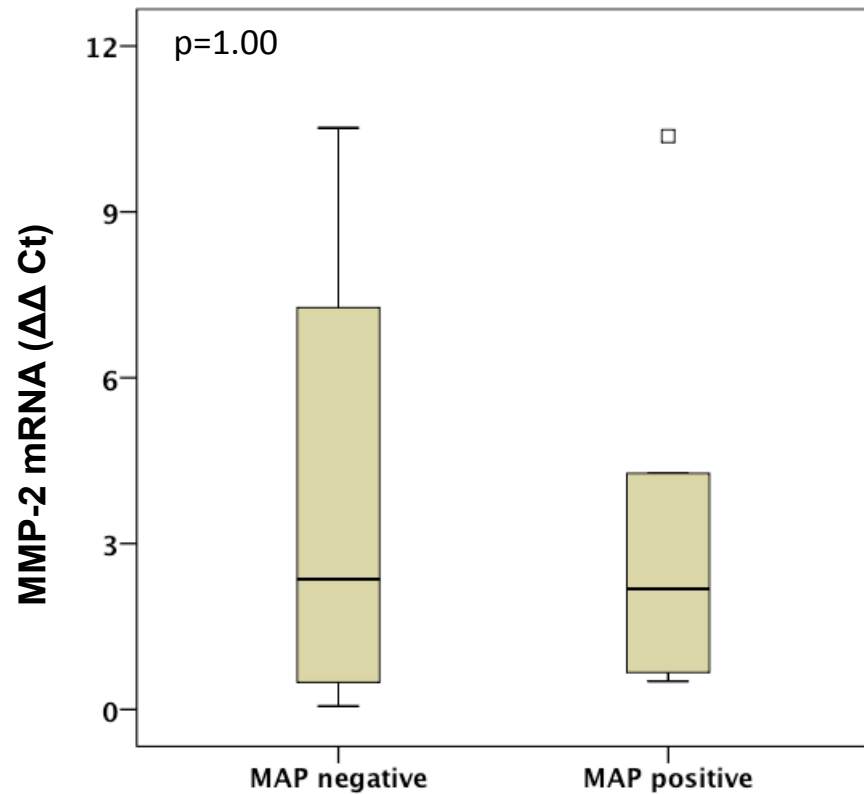

Supplement: Additional file 1 — Expression of MMP-1 and MMP-2 in UC patients without corticosteroids with respect to the presence of MAP DNA (n = 41). mRNA results were determined by RT-PCR. MMP-1 and MMP-2 gene expression was not significantly different in steroidfree UC patients with intestinal MAP detection compared to those without. [file 1471-230X-11-34-S1.PDF]

**MMP-7 mRNA ( $\Delta\Delta$  Ct)**

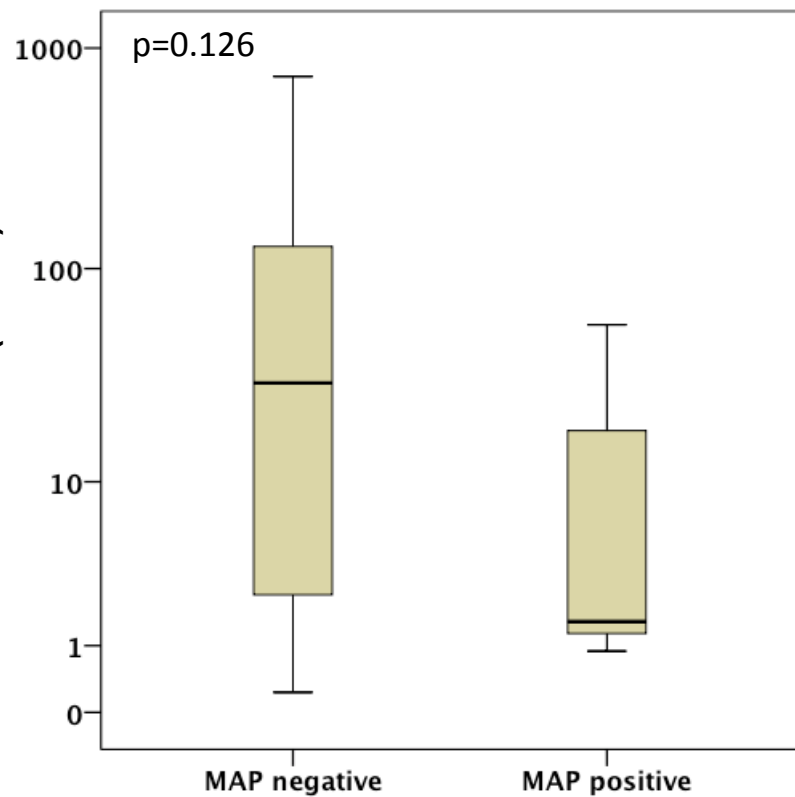

**MMP-9 mRNA ( $\Delta\Delta$  Ct)**

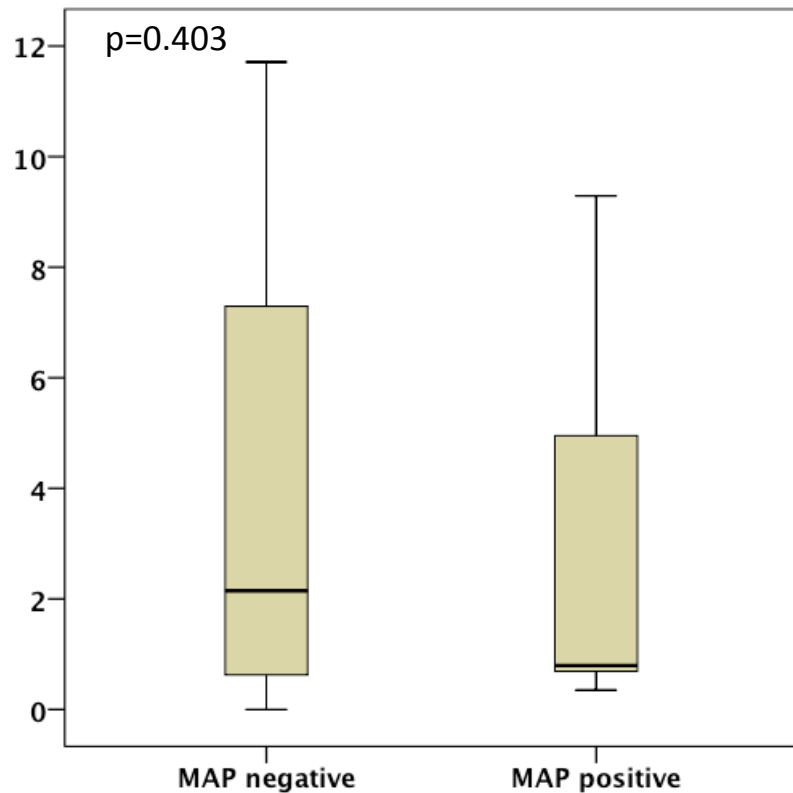

Supplement: Additional file 2 — Expression of MMP-7 and MMP-9 in UC patients without corticosteroids with respect to the presence of MAP DNA (n = 41). mRNA results were determined by RT-PCR. MMP-7 and MMP-9 gene expression was not significantly different in steroidfree UC patients with intestinal MAP detection compared to those without. [file 1471-230X-11-34-S2.PDF]

**MMP-13 mRNA ( $\Delta\Delta$  Ct)**

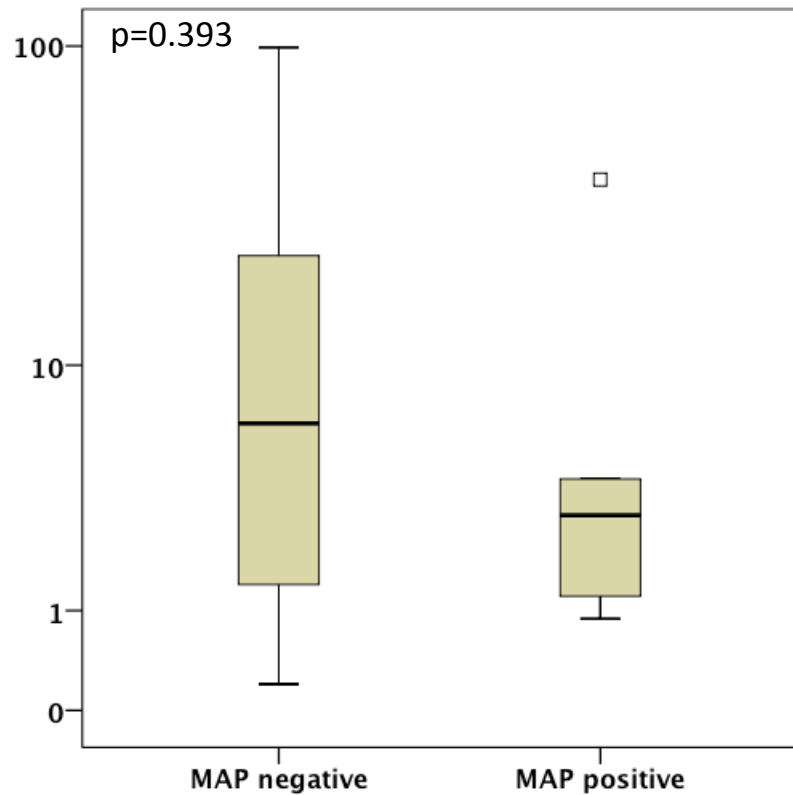

**MMP-19 mRNA ( $\Delta\Delta$  Ct)**

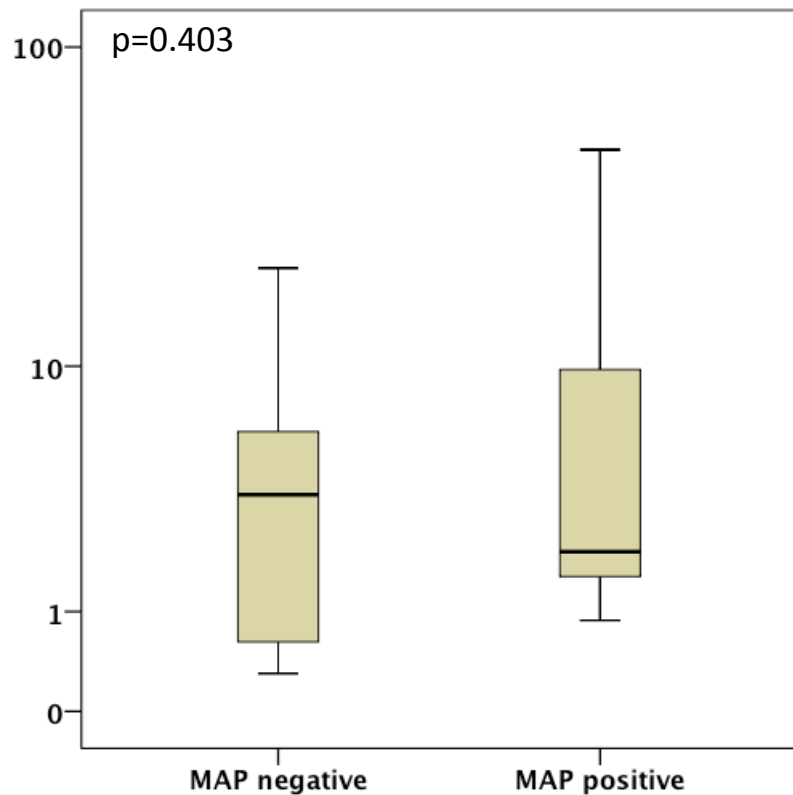

Supplement: Additional file 3 — Expression of MMP-13 and MMP-19 in UC patients without corticosteroids with respect to the presence of MAP DNA (n = 41). mRNA results were determined by RT-PCR. MMP-13 and MMP-19 gene expression was not significantly different in steroidfree UC patients with intestinal MAP detection compared to those without. [file 1471-230X-11-34-S3.PDF]

**MMP-28 mRNA ( $\Delta\Delta$  Ct)**

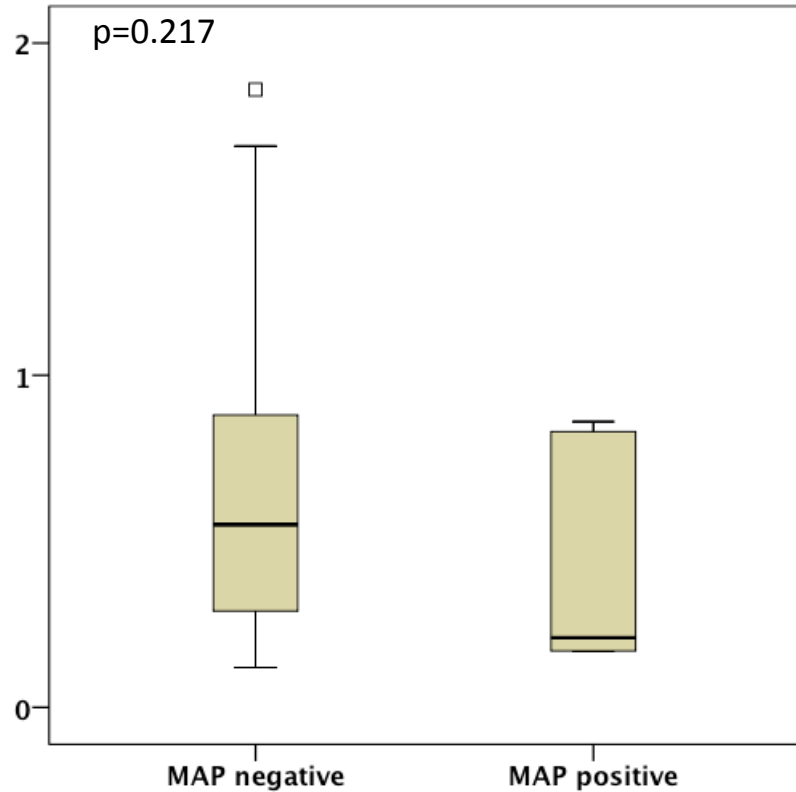

**TNF- $\alpha$  mRNA ( $\Delta\Delta$  Ct)**

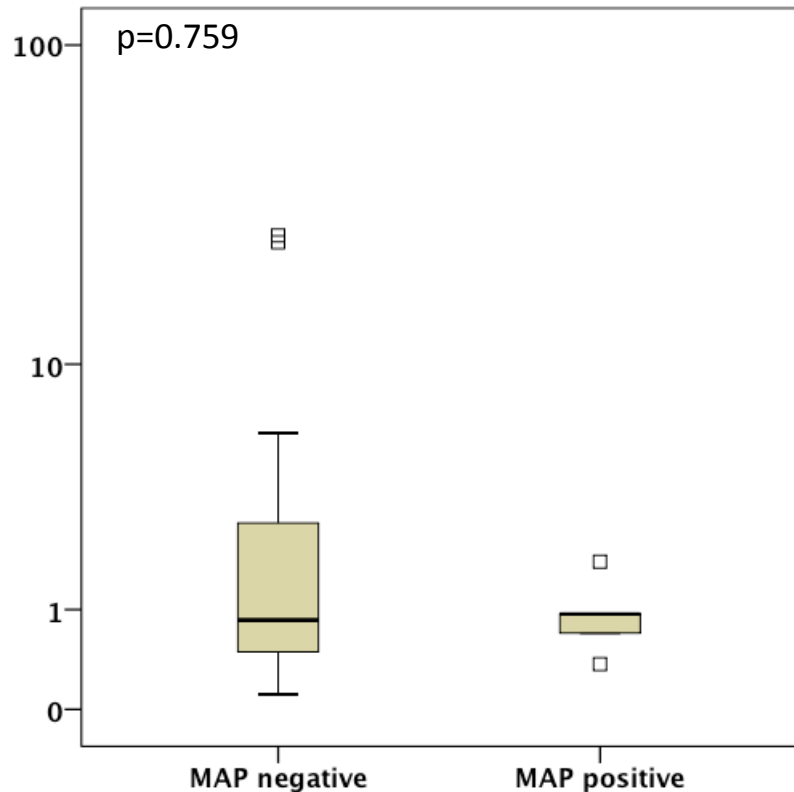

Supplement: Additional file 4 — Expression of MMP-28 and TNF-α in UC patients without corticosteroids with respect to the presence of MAP DNA (n = 41). mRNA results were determined by RT-PCR. MMP-28 and TNF-α gene expression was not significantly different in steroidfree UC patients with intestinal MAP detection compared to those without. [file 1471-230X-11-34-S4.PDF]
